# Comparative genomics reveals new evolutionary and ecological patterns of selenium utilization in bacteria

**DOI:** 10.1038/ismej.2015.246

**Published:** 2016-01-22

**Authors:** Ting Peng, Jie Lin, Yin-Zhen Xu, Yan Zhang

**Affiliations:** 1 Key Laboratory of Nutrition and Metabolism, Institute for Nutritional Sciences, Shanghai Institutes for Biological Sciences, Chinese Academy of Sciences, University of Chinese Academy of Sciences, Shanghai, PR China

## Abstract

Selenium (Se) is an important micronutrient for many organisms, which is required for the biosynthesis of selenocysteine, selenouridine and Se-containing cofactor. Several key genes involved in different Se utilization traits have been characterized; however, systematic studies on the evolution and ecological niches of Se utilization are very limited. Here, we analyzed more than 5200 sequenced organisms to examine the occurrence patterns of all Se traits in bacteria. A global species map of all Se utilization pathways has been generated, which demonstrates the most detailed understanding of Se utilization in bacteria so far. In addition, the selenophosphate synthetase gene, which is used to define the overall Se utilization, was also detected in some organisms that do not have any of the known Se traits, implying the presence of a novel Se form in this domain. Phylogenetic analyses of components of different Se utilization traits revealed new horizontal gene transfer events for each of them. Moreover, by characterizing the selenoproteomes of all organisms, we found a new selenoprotein-rich phylum and additional selenoprotein-rich species. Finally, the relationship between ecological environments and Se utilization was investigated and further verified by metagenomic analysis of environmental samples, which indicates new macroevolutionary trends of each Se utilization trait in bacteria. Our data provide insights into the general features of Se utilization in bacteria and should be useful for a further understanding of the evolutionary dynamics of Se utilization in nature.

## Introduction

Selenium (Se) is an important trace element for many organisms in the three domains of life; yet, it is required only in small amounts. This element is known primarily for its functions in redox homeostasis, and is recognized as a promising cancer chemo-preventive agent. In addition, Se is required for mammalian development, male reproduction and immune function (Rayman, 2000; Hatfield *et al.*, 2006). To date, three biological forms of Se have been identified: (i) selenocysteine (Sec, the 21st amino acid), the major biological form of Se, which is co-translationally inserted into selenoproteins by recoding the UGA codon (Böck *et al.*, 1991; Stadtman, 1996; Hatfield and Gladyshev, 2002); (ii) 5-methylaminomethyl-2-selenouridine (mnm^5^Se^2^U or SeU), which is located at the wobble position of the anticodons of some prokaryotic tRNAs and may help base pair discrimination and/or improve translation efficiency (Ching *et al.*, 1985; Wittwer and Ching, 1989); (iii) a Se-containing cofactor (Se-cofactor) used by certain molybdenum-containing enzymes in both bacteria and archaea (Haft and Self, 2008; Zhang *et al.*, 2008).

The decoding of UGA as Sec is an intriguing example of alternative genetic decoding phenomenon. Alterations in genetic decoding are expected to exist in almost all organisms (Baranov *et al.*, 2015). Many mRNAs have evolved special elements to alter the meaning of specific codons or to result in ribosomal frameshifting or even translational bypassing (Atkins and Baranov, 2010; Chin, 2014). The mechanism of Sec biosynthesis and its insertion into proteins has generally been elucidated in all three domains of life (Low and Berry, 1996; Böck, 2000; Thanbichler and Böck, 2002; Allmang *et al.*, 2009). In bacteria, this process requires an in-frame UGA codon, a Sec insertion sequence (SECIS) element that is a hairpin structure within the selenoprotein mRNA immediately downstream of the Sec-encoding UGA codon, and several *trans*-acting factors including Sec synthase (SelA), Sec-specific elongation factor (SelB), Sec-specific tRNA^[Ser]Sec^ (a unique tRNA whose anticodon matches the UGA codon) and selenophosphate synthetase (SelD). In eukaryotes and archaea, the biosynthesis of Sec uses a similar mechanism as in bacteria. However, additional genes such as genes encoding the eukaryotic/archaeal Sec synthase, O-phosphoseryl-tRNA^[Ser]Sec^ kinase and SECIS binding protein SBP2 are needed for incorporation of Sec into protein (Copeland and Driscoll, 2001; Xu *et al.*, 2007; Squires and Berry, 2008). In bacteria, the entire Sec-decoding trait and selenoprotein genes could be laterally transferred between evolutionarily distant organisms to extend Se utilization (Romero *et al.*, 2005; Zhang *et al.*, 2006); however, such event could not be detected in multicellular eukaryotes such as animals.

In many prokaryotes, the product of SelD protein, selenophosphate, is also used for the biosynthesis of SeU. The 2-selenouridine synthase (YbbB) is needed during SeU utilization (Wolfe *et al.*, 2004). In addition, a new SelD-based Se utilization form was reported in some bacteria, in which Se might be used as a cofactor by molybdenum-containing hydroxylases via two gene products whose function is unclear as yet: YqeB and YqeC (Haft and Self, 2008; Zhang *et al.*, 2008). Thus, it appears that each known Se utilization trait has unique genes, whereas SelD is considered as a common signature for Se utilization (Romero *et al.*, 2005; Zhang *et al.*, 2008).

In the recent decade, the majority of studies on Se focused on investigation of new genes and pathways involved in Se metabolism and the function of selenoproteins. In spite that several comparative genomics analyses of components of Se utilization traits in completely sequenced bacterial genomes have provided evidence for a highly diverse pattern of species that use Se (Romero *et al.*, 2005; Zhang *et al.*, 2006, 2008; Zhang and Gladyshev, 2010b; Lin *et al.*, 2015), it is still unclear how this trace element is used by a subset of organisms. A more puzzling question is whether the evolution of Se utilization could be influenced by various ecological conditions. With the rapid increase in the number of sequenced genomes in recent years, it would be interesting to determine the general utilization trend of different Se forms and the relationship between environmental conditions and Se utilization.

In this study, we applied comparative genomics approaches to more than 5200 sequenced bacterial genomes to investigate Se utilization in this domain. First, we analyzed the distribution of key components involved in different Se utilization traits and generated a large phylogeny map of Se utilization in bacteria. We also identified a subset of organisms that had *selD* gene but lacked any of the known Se traits, implying that a new SelD-based Se utilization pathway is present in these organisms. Further analysis of the whole set of selenoproteins (selenoproteomes) of all Sec-utilizing organisms showed the presence of a new selenoprotein-rich phylum and more selenoprotein-rich organisms in bacteria. Finally, analysis of environmental factors of all organisms revealed that different Se traits may favor specific ecological conditions such as habitat, oxygen concentration and temperature. As a whole, these data provide a better understanding of the general trends of Se utilization and evolution in bacteria.

## Materials and methods

### Genomic sequences and resources

Both completely and almost completely sequenced bacterial genomes were downloaded from the ftp site of National Center for Biotechnology Information (NCBI) (ftp://ftp.ncbi.nlm.nih.gov/genomes/). A total of 5207 genomes were retrieved (as of January 2014). Information about environmental and other factors (such as habitat, oxygen requirement, optimal growth temperature and Gram staining) associated with these organisms was acquired from either NCBI or Genomes OnLine Database (GOLD) (Reddy *et al.*, 2015).

### Identification of Se utilization traits in different organisms

To analyze the occurrence of different Se traits, we used *Escherichia coli* SelD, SelA and SelB sequences as queries to search for components of the Sec trait (Zhang *et al.*, 2006), SelD and YbbB for the SeU trait (Wolfe *et al.*, 2004; Zhang *et al.*, 2006), and SelD, YqeB and YqeC for the Se-cofactor trait (Zhang *et al.*, 2008). TBLASTN (Altschul *et al.*, 1990) was initially used to identify genes encoding homologs with a cutoff E-value of 0.01 and the alignment coverage of at least 30%. Orthologous proteins were defined using the conserved domain database (Pfam or CDD) (Bateman *et al.*, 2002; Marchler-Bauer *et al.*, 2015) and bidirectional best hits (Wolf and Koonin, 2012). If necessary, orthologs were also confirmed by genomic location or phylogenetic analyses. The distribution of SelD and the three known Se utilization traits in different bacterial taxa was presented by using the online Interactive Tree Of Life (iTOL) tool (Letunic and Bork, 2007, 2011) based on a recently developed phylogenetic tree of life (Ciccarelli *et al.*, 2006).

### Multiple sequence alignment and phylogenetic analysis

Standard approaches were used to reconstruct phylogenetic trees of each component of Se utilization traits. Multiple sequence alignments were performed using CLUSTALW (Thompson *et al.*, 1994) with default parameters. Phylogenetic trees of protein families were reconstructed by PHYLIP programs (Felsenstein, 1989) using neighbor-joining method, and were further evaluated by MrBayes (Bayesian estimation of phylogeny) tool (Ronquist and Huelsenbeck, 2003). The vector graphics editor Inkscape software (version 0.91) (Minatani, 2015) was used to further refine the fonts and colors of the phylogenetic trees.

### Identification of selenoproteomes in all Sec-utilizing organisms

We collected representative sequences for all bacterial selenoprotein families identified or predicted before (Kryukov and Gladyshev, 2004; Zhang and Gladyshev, 2005, 2008, 2010a; Zhang *et al.*, 2005). These sequences were then used to search against the genomic sequences of all Sec-utilizing organisms for selenoprotein homologs with TBLASTN (*e*-value ⩽0.01). The redundant selenoprotein sequences were removed using a custom script program. The presence of a putative Sec-encoding UGA codon and a downstream SECIS element was also analyzed using the bSECISearch tool (Zhang and Gladyshev, 2005).

### Metagenomic analysis of Se utilization

A total of 60 bacterial metagenomic data sets derived from different ecological environments (marine, freshwater, terrestrial and host-associated; 15 data sets for each) were retrieved from the Joint Genome Institute Metagenome/Integrated Microbial Genomes (JGI-M/IMG) database (Markowitz *et al.*, 2012). To identify each Se utilization trait in different environmental samples, we used a strategy that was similar to what we had done for sequenced organisms (see above). In each sample, if the occurrence of genes involved in the same Se utilization trait was different, the smallest number was used to represent the frequency of the corresponding Se trait. The recombinase A (*recA*) gene, which is a highly conserved housekeeping gene and has been widely used in many comparative metagenomic studies (Handelsman, 2004; Wu *et al.*, 2011), was used for normalization of the occurrence of each gene in samples.

## Results

### Generation of the largest map of Se utilization in bacteria

**Figure 1:**
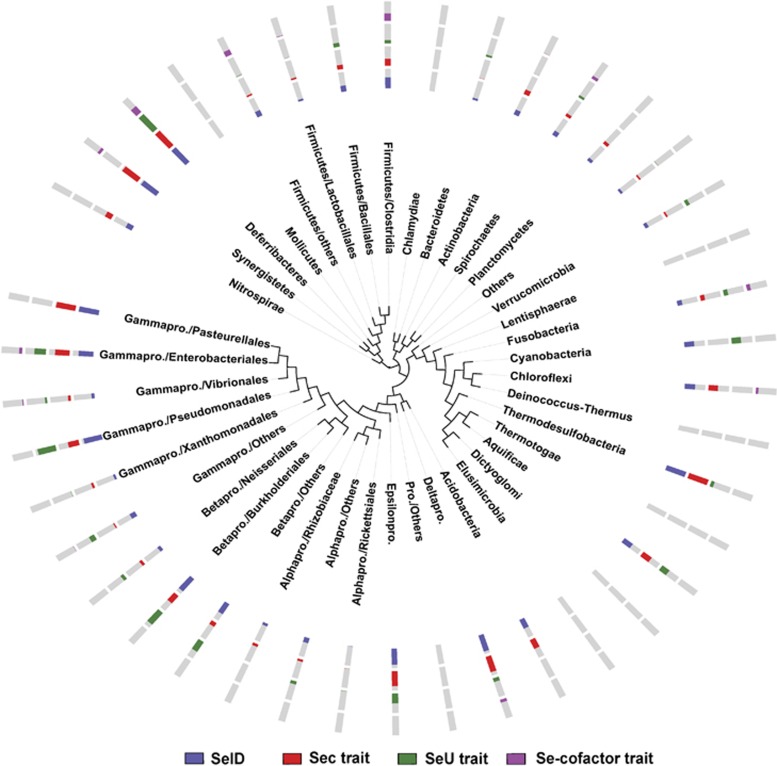
Distribution of SelD and known Se utilization traits in bacteria. The phylogenetic tree is simplified to only show major bacterial taxa and branches. The four tracks (circles) around the tree (from inside to outside) represent the distribution patterns of SelD, Sec trait, SeU trait and Se-cofactor trait, respectively. The length of the colored section of each column represents the percentage of organisms that have either SelD or the corresponding Se trait among all sequenced organisms in this phylum: purple, SelD; red, Sec trait; green, SeU trait; pink, Se-cofactor trait; grey, the rest of sequenced organisms. pro. stands for proteobacteria.

Very recently, we have analyzed the distribution of key components involved in each Se utilization machinery, including Sec (SelD/SelA/SelB), SeU (SelD/YbbB) and Se-cofactor (SelD/YqeB/YqeC) traits, in more than 2000 bacterial genomes (Lin *et al.*, 2015). In this study, we extended such analysis to more than 5200 bacterial genomes, which has generated the largest and the most precise Se utilization map in bacteria thus far. Figure 1 represents the distribution patterns of SelD and three Se utilization traits in different bacterial phyla or clades (details of the occurrence of SelD and other key components involved in each Se utilization trait are shown in Supplementary Table S1).

It has been proposed that SelD protein was required for all Se utilization traits, which defined the overall Se utilization. In this study, except for phyla containing one or two sequenced genomes (such as Dictyoglomi, Elusimicrobia and Lentisphaerae), SelD was detected in almost all bacterial lineages with the exception of a small number of phyla or clades such as Chlamydiae, Mollicutes, Deinococcus-Thermus and Thermotogae. This implies that Se utilization is an ancient trait that once was common to almost all species in bacteria. However, only 1754 organisms (33.7% of all sequenced bacteria) were detected to contain SelD, suggesting that most species have lost the ability to use this micronutrient over the long process of evolution. This is consistent with previous observations (Zhang *et al.*, 2006; Lin *et al.*, 2015).

**Figure 2:**
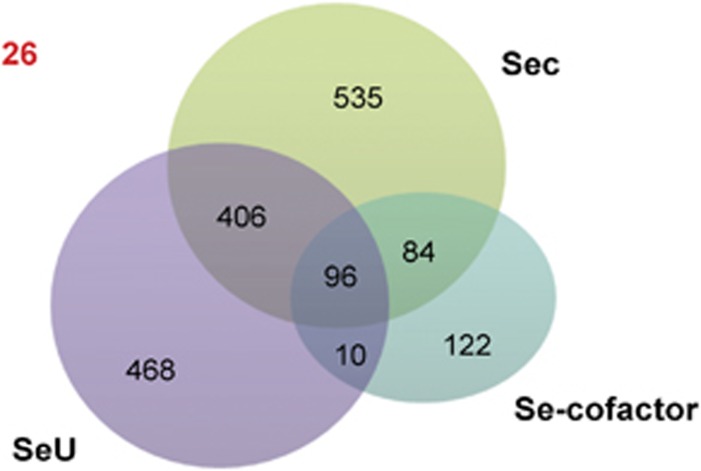
Venn diagram representation of the distribution of Se utilization traits in bacteria. The number of organisms containing the corresponding Se traits is indicated. The number of organisms that contain orphan SelD is highlighted in red.

Among all sequenced bacterial genomes, we identified 1121 Sec-utilizing (21.5%), 980 SeU-utilizing (18.8%) and 312 Se-cofactor-utilizing (6.0%) organisms. SelB has been suggested to be the signature of the Sec and YbbB of the SeU traits (Romero *et al.*, 2005). Our data showed a perfect consistency between these two genes and the occurrence of Sec or SeU traits. Because of the common component SelD, significant overlaps could be observed between different Se utilization traits (Figure 2). For example, 96 organisms were found to possess all known Se utilization traits. Hypergeometric test suggested a significant relationship between any two of the three Se utilization traits (*P-*value <0.05). All these results were consistent with our previous assumption that the presence of one Se trait may be beneficial to acquisition of others, probably partially due to the presence of SelD (Zhang *et al.*, 2006; Lin *et al.*, 2015).

Some organisms were found to have highly similar homologs of certain proteins (for example, SelA, YbbB, YqeB or YqeC), but lack a complete Se utilization trait (details are shown in Supplementary Table S1). Considering that the majority of them have not been fully sequenced or assembled, the possibility that other known genes were not sequenced could not be neglected. It is also possible that some of these homologs are involved in Se-independent processes in certain organisms. The hypothesis that SelA may have additional function has been proposed before (Zhang *et al.*, 2006). Very recently, it was suggested that an alternative enzymatic function might be hidden in the SelA protein in *Helicobacter pylori* that does not use Se (Cravedi *et al.*, 2015). In addition, only the co-occurrence of YqeB and YqeC proteins could be used as a signature for the Se-cofactor utilization trait, implying that each of them may participate in some processes that are unrelated to Se (Zhang *et al.*, 2008).

### Identification of organisms containing orphan SelD

Previously, we have noticed the presence of very few organisms that had orphan SelD (containing SelD but lacking any other known components of Se utilization traits and selenoprotein genes) (Lin *et al.*, 2015). With the largest number of sequenced bacterial genomes retrieved in this study, we identified at least 26 orphan SelD-containing organisms (Table 1). These organisms belong to several evolutionarily distant bacterial phyla or subdivisions, such as *Actinopolyspora iraqiensis IQ-H1* (Actinobacteria), *Brevundimonas sp. BAL3* and *Methylobacterium nodulans* (Alphaproteobacteria), *Xenorhabdus bovienii* and *X. nematophila* (Gammaproteobacteria/Enterobacteriales) and *Prochlorococcus sp. W7*~*W9* (Cyanobacteria). It is most likely that a novel and unknown SelD-based Se utilization trait is present in at least some of these organisms. On the other hand, the possibility that some of these *selD* genes are either pseudogenes or involved in non-Se-related processes could not be fully excluded.

To investigate possible genes that might be involved in this unknown SelD-related trait, we examined 10 genes upstream and downstream of *selD* in these organisms. Three genes were chosen as SelD-related candidates due to their genomic locations close to *selD* in at least three organisms belonging to different phyla, including genes encoding an isochorismatase-like protein (pfam00857), ABC transporter-related ATPase (pfam00005) and a cysteine desulfurase-like (pfam00266) protein (Supplementary Figure S1). Specific functions of these proteins are not clear yet. Future experiments are needed to verify the expression of orphan *selD* genes in the corresponding organisms and to study the relationship between these proteins and SelD or Se metabolism.

### Identification of new horizontal gene transfer events for all Se utilization traits

Previous comparative studies of Se utilization have reported several horizontal gene transfer (HGT) events for both Sec and SeU traits, such as HGTs of the entire Sec utilization pathway observed between *Treponema denticola* (Spirochaetes) and *Photobacterium profundum* (Gammaproteobacteria), as well as between *Pseudomonadale* species (Gammaproteobacteria) and Betaproteobacteria (Romero *et al.*, 2005; Zhang *et al.*, 2006). In this study, we reconstructed the phylogenetic trees for the key components of each known Se trait based on thousands of bacterial species. In spite that most branches were consistent with the evolutionary relationship among bacterial species, we identified new HGT events for each Se utilization trait, including the Se-cofactor trait that had not been reported so far.

Table 2 shows the HGT events that may have happened very recently, which could be supported by the widespread occurrence of the Se trait in many species of the donor-containing phyla and the absence of the same trait in all or almost all sister species of the recipient organism. For example, among all sequenced *Marinobacter* species (Gammaproteobacteria/Alteromonadales), *M. daepoensis DSM 16072* was the only organism that contains the Sec trait, which might have acquired from *Pseudomonas* species (Gammaproteobacteria/Pseudomonadales). Moreover, in the majority of both donor and recipient organisms, the key genes (including *selD*) involved in the corresponding Se utilization traits as well as the only selenoprotein gene (formate dehydrogenase alpha subunit) in those Sec-utilizing organisms are either very close or even organized in single operons.

**Figure 3:**
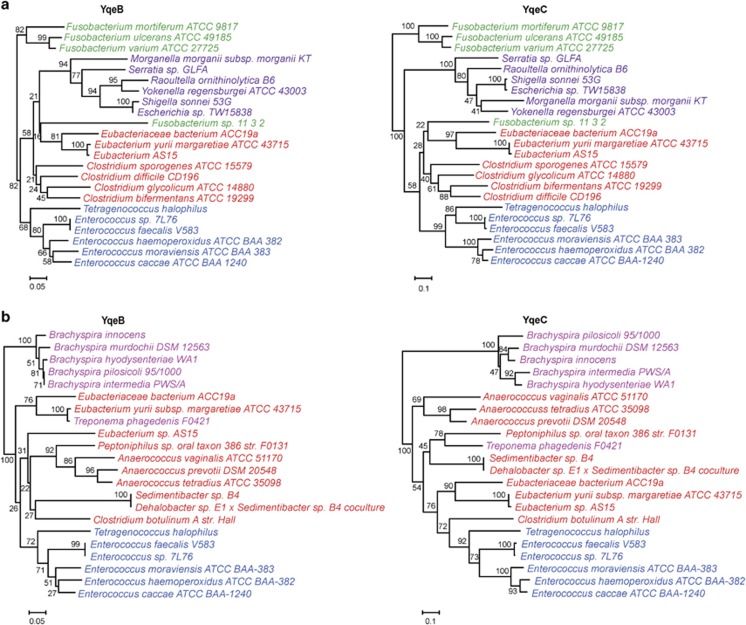
New HGT events revealed by phylogenetic analysis of YqeB and YqeC sequences. Organisms from different phyla or clades are shown in different colors. (**a**) HGT between Firmicutes/Clostridia and *Fusobacterium sp. 11_3_2* and (**b**) HGT between Firmicutes/Clostridia and *T. phagedenis F0421*. Red: Firmicutes/Clostridia, blue: Firmicutes/Lactobacillales, purple: Gammaproteobacteria/Enterobacteriales, green: Fusobacteria, pink: Spirochaetes. The branch lengths and bootstrap values are also shown.

Here, we identified HGT events for the Se-cofactor trait in nature even though it was very rare. The phylogenetic tree topologies of both YqeB and YqeC revealed that the *Fusobacterium sp. 11_3_2* (Fusobacteria) sequences were within the Firmicutes/Clostridia node, and not within the Fusobacteria node as expected by vertical descent (Figure 3a). Similar HGT was also observed for *Treponema phagedenis F0421* (Spirochaetes), which also acquired the Se-cofactor trait from Firmicutes/Clostridia (Figure 3b). Thus, HGT events appeared to have contributed to the evolution of all Se utilization traits. However, so far we could not identify one HGT event for the co-transfer of more than one Se utilization traits.

One previous hypothesis was that in the presence of SelD that has been used by one Se trait, acquisition of an additional Se trait via HGT might be easier during evolution (Zhang *et al.*, 2006). Here, more HGT events were observed in organisms that lack any other Se utilization traits (Table 2). This implies that the pre-existing *selD* gene is not a strong factor that could influence the occurrence of HGT, although less Se-related genes are needed to be laterally transferred from other organisms. However, considering that the HGT events observed for all Se utilization traits are very rare, the advantage of an already existing *selD* gene for acquiring other Se traits by HGT is not clear yet.

### Identification of selenoproteomes in all sequenced Sec-utilizing organisms

As mentioned above, Sec is the major biological form of Se and is incorporated into selenoproteins to participate in a variety of redox and metabolic processes. To better understand the utilization and function of Se, it would be important to characterize the selenoproteomes in different organisms. Here, we have collected all known and previously predicted prokaryotic selenoprotein families reported to date (∼90 families or subfamilies) and used representative sequences for each of them to search for Sec-containing homologs in all Sec-utilizing organisms (see Materials and methods). The number of examined organisms was eight times more than previous studies (Zhang and Gladyshev, 2010b).

**Figure 4:**
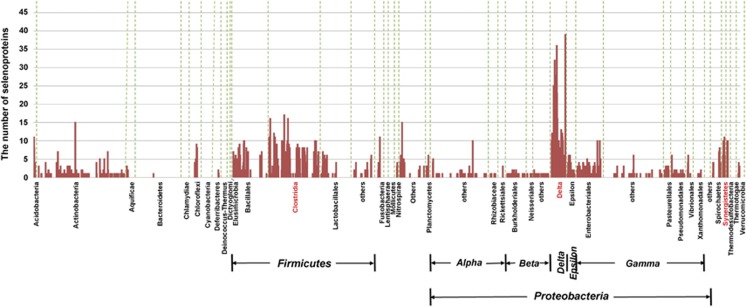
Occurrence and composition of selenoproteomes in bacteria. The majority of selenoprotein-rich organisms (containing at least six selenoprotein genes) belong to three phyla, which are highlighted in red: Deltaproteobacteria, Firmicutes/Clostridia and Synergistetes.

The occurrence and size of selenoproteomes in different bacterial phyla or clades are shown in Figure 4 (details are shown in Supplementary Table S2). Two bacterial phyla, Deltaproteobacteria and Firmicutes/Clostridia, have been previously considered as selenoprotein-rich phyla in which the majority of sequenced species were selenoprotein-rich organisms (that is, containing at least six selenoprotein genes as defined before) (Zhang *et al.*, 2006; Zhang and Gladyshev, 2009, 2010b). On the basis of current results, much more selenoprotein-rich organisms could be identified in these two phyla, including several new organisms that have more than 30 selenoprotein genes (Supplementary Table S2). To date, the largest selenoproteome in sequenced bacteria has been found in *Syntrophobacter fumaroxidans* (Deltaproteobacteria), which contains at least 39 selenoprotein genes (Zhang and Gladyshev, 2010b). Interestingly, we identified a new selenoprotein-rich phylum: Synergistetes. In this phylum, 13 out of 14 sequenced organisms (92.9%) could use Sec and at least 10 organisms (71.4%) were selenoprotein-rich organisms (Figure 4). It should be noted that a small number of Sec-utilizing organisms appeared to lack known selenoprotein genes, most likely due to their incomplete genomes. In addition, the possibility that new selenoprotein genes are present in some of these organisms could not be excluded. Further investigation of all known selenoprotein families revealed that the majority of them could be identified in currently sequenced bacteria. Distribution of sequences and organisms for the top 20 selenoprotein families is shown in Table 3 (more details are shown in Supplementary Table S3). Formate dehydrogenase alpha subunit and SelD were the most widespread selenoproteins in bacteria, which is consistent with previous observation (Zhang *et al.*, 2006). We also measured the fraction of selenoprotein genes in all completely sequenced Sec-utilizing organisms. No significant correlation could be observed between the total number of selenoprotein genes and that of all genes annotated in the corresponding genomes (Supplementary Figure S2). Thus, our data greatly extended previous analysis by examining additional sequenced genomes and provided new information on the complex evolutionary process of selenoproteins in bacteria.

### Re-investigation of the relationship between Se utilization and environmental factors

Previously, by using a limited number of organisms, we tried to analyze the relationship between the Sec and/or the SeU traits and different factors (for example, habitat, oxygen requirement, optimal temperature, geographical location, Gram strain and GC content). It was suggested that the Sec utilization trait might favor anaerobic and/or hyperthermic conditions, whereas the SeU trait might favor aerobic and mesophilic conditions (Zhang *et al.*, 2006; Zhang and Gladyshev, 2010b). Considering the number and distribution bias of sequenced bacterial genomes at that time, it is necessary to recheck such relationship using much more sequenced organisms available now.

**Figure 5:**
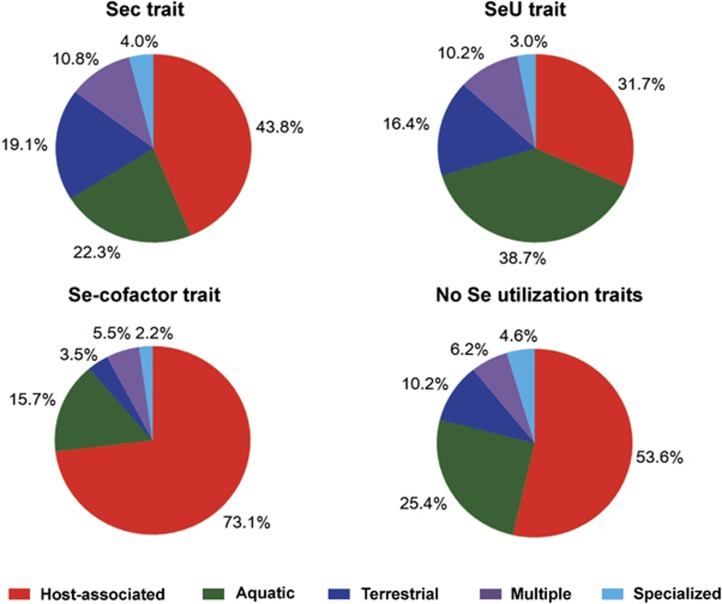
Relationship between Se utilization traits and habitat. Five types of habitat were analyzed, including host-associated, aquatic, terrestrial, multiple and specialized.

**Figure 6:**
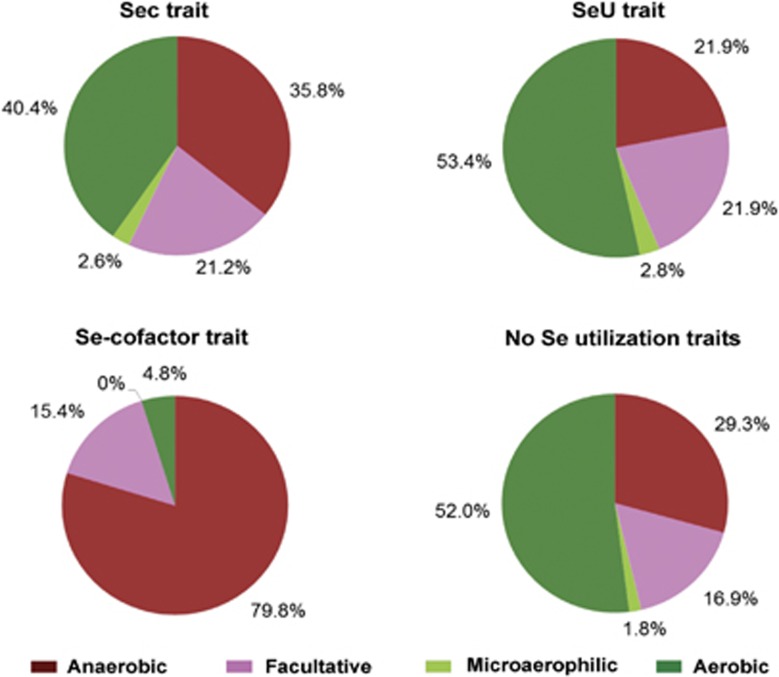
Relationship between Se utilization traits and oxygen requirement. Four types of oxygen requirement were analyzed, including anaerobic, facultative, microaerophilic and aerobic.

In this study, we collected the information about ecological environments and some other factors for all organisms, and analyzed the contribution of each of these factors for organisms containing Sec, SeU and/or Se-cofactor traits. First, we found that both Sec and Se-cofactor traits favored a host-associated condition, whereas organisms possessing the SeU trait were aquatic species that were mostly isolated from sea or freshwater (Figure 5). Second, in contrast with the previous observation (Zhang *et al.*, 2006), oxygen requirement analysis revealed that Sec-utilizing organisms appeared to favor both aerobic and anaerobic conditions (40.4% and 35.8%, respectively; Figure 6). Thus, our previous hypothesis that anaerobic condition was a factor promoting the use of Sec might be biased, most likely due to the insufficient number of genomes of aerobic organisms at that time. With a significant increase in the number of sequenced aerobic species in the recent decade, our results should be more reasonable to show a general trend of Sec utilization in bacteria. However, the fact that more than three-fourths of selenoprotein-rich organisms (78.3%) were anaerobic species suggested a somewhat more complex relationship between selenoproteins and low oxygen level. The majority of SeU-utilizing organisms (53.4%) favored aerobic environments (Figure 6), which is consistent with our previous observation. On the other hand, almost four fifths (79.8%) of the Se-cofactor-utilizing organisms lived in anaerobic environment (Figure 6). Thus, it appears that anaerobic conditions could significantly promote the use of the Se-cofactor trait, which has never been reported before. A more detailed analysis of the correlation between oxygen requirement and different Se traits is shown in Supplementary Figure S3A. Finally, temperature seemed to be an important factor that could affect different Se utilization traits. The Sec trait favored thermophilic conditions, whereas psychrophilic bacteria appeared to prefer the SeU trait (Supplementary Figure S3B), which basically agreed with previous results (Zhang *et al.*, 2006). No significant correlation could be observed between temperature and the Se-cofactor trait. Other factors, such as geographical location, Gram strain and GC content, had no significant effect on the evolution of Se utilization.

### Metagenomic analysis of Se utilization traits

To further verify the relationship between Se utilization patterns and the habitat/environmental preferences of bacteria, we performed a comparative metagenomic analysis of Se utilization in 60 bacterial communities from different habitats or environments: aquatic (marine and freshwater), terrestrial and host-associated. Sample details are shown in Supplementary Table S4.

**Figure 7:**
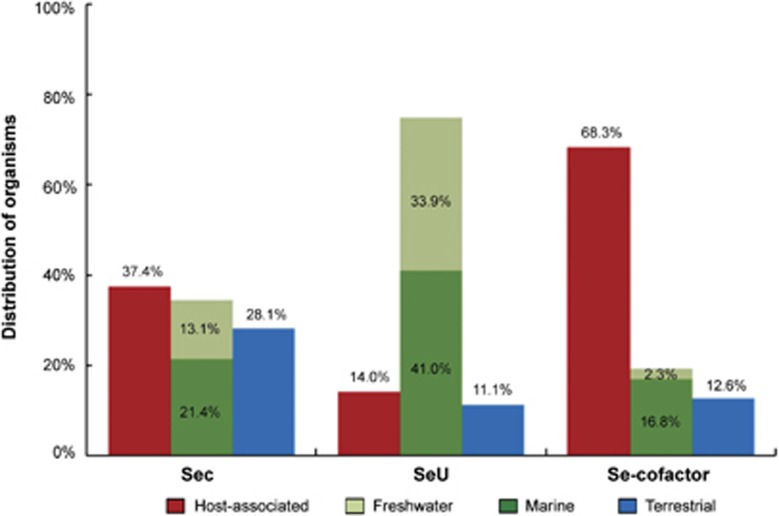
Metagenomic analysis of the relationship between Se utilization and different environments. Samples were collected from four types of habitat: host-associated, marine (aquatic), freshwater (aquatic) and terrestrial.

Analysis of the occurrence of *selD* gene revealed that it might not be essential for the majority of organisms in almost all environmental samples examined here, which is generally consistent with the above observation (Supplementary Figure S4). Based on identification of the occurrence of genes for each Se utilization trait in each environmental sample data set, we found that the Sec utilization trait was more frequently present in host-associated and aquatic (especially marine) habitats, whereas the SeU and Se-cofactor traits were significantly enriched in aquatic (either marine or freshwater) and host-associated bacterial communities, respectively (Figure 7). This is also consistent with what we have observed in sequenced genomes. Therefore, our results provide new insights into the global trend in microbial Se utilization in different ecological environments.

## Discussion

The importance of Se in the physiology of both prokaryotes and eukaryotes has been well established (Rayman, 2000; Hatfield *et al.*, 2006). Although much effort has been placed on characterizing pathways that Se is involved in and the function of selenoproteins (Forchhammer and Böck, 1991; Kryukov *et al.*, 2003; Zhang and Gladyshev, 2008, 2009; Zhang *et al.*, 2008), the evolution and ecology of Se utilization traits remains largely unknown. Some considerations about the evolutionary patterns of different Se traits and selenoprotein families have previously been proposed (Zhang *et al.*, 2006; Castellano, 2009; Zhang and Gladyshev, 2009, 2010b). However, due to much less genomic resources available at that time, it is unclear whether these observations or hypotheses could correctly reflect the general distribution and evolutionary dynamics of Se utilization over the whole bacterial domain. In the present study, we greatly extended such analysis for all known Se utilization traits to more than 5200 bacterial species, which was eight times larger than previous analysis (Zhang and Gladyshev, 2010b). Our data provide the largest and most comprehensive view of Se utilization in bacteria. Such strategy and resources collected here can be easily extended to analyze the utilization and evolution of many other micronutrients such as metals.

Comparative genomics analysis of known Se utilization traits revealed that Se is an important element for a variety of organisms in almost all bacterial phyla. However, among all sequenced bacterial organisms, only one-third of organisms had at least one Se trait, implying that most bacterial lineages lost the ability to use this element. Previous hypothesis that different Se traits could affect evolution of each other (Zhang *et al.*, 2006; Lin *et al.*, 2015) has been further confirmed by analyzing more bacterial genomes.

As SelD is a common feature for all Se utilization pathways, identification of several species that possess orphan *selD* gene raised the possibility of a new SelD-related Se utilization trait in these organisms. By examining several genes upstream and downstream of *selD* gene in each of those genomes, three of them were considered as candidates that might be functionally related to SelD, including isochorismatase-like, ABC transporter-related ATPase and cysteine desulfurase-like proteins. Future challenges would be to experimentally verify the new SelD-dependent Se form and identify the function of these genes.

It is known that HGT events can contribute to the evolution of a variety of biological processes, including Se utilization (Romero *et al.*, 2005; Zhang *et al.*, 2006). Although HGT of the entire Se utilization trait is difficult, we observed additional cases besides the HGT events previously reported for the Sec and SeU traits. For the first time, we identified HGT for the Se-cofactor trait, which has never been reported before. Moreover, identification of more HGT events in the organisms that lack any other Se traits suggests that an already existing *selD* gene used by one Se trait might not be strongly needed by HGT of additional Se traits in certain organisms.

There is no doubt that, in this study, identification of selenoproteomes in all Sec-utilizing organisms has generated the largest data set of selenoproteins in this domain of life. These data revealed new organisms that have a large number of selenoproteins and a new selenoprotein-rich phylum: Synergistetes, which provide novel insights into the evolution of selenoproteins in bacteria. In addition, newly identified selenoprotein-rich organisms may have far-reaching implications for their potential use for the development of Se-enriched bio-products. For example, several Se-enriched algae (such as *Palmaria Palmata*) have been regarded as the best alternatives to cereals in food and feed (Maehre *et al*, 2014). In the future, it would be valuable to investigate the evolutionary dynamics of different selenoprotein families and the complex crosstalk among them.

An additional significant contribution of this work is that re-investigation of the relationship between environmental factors and Se utilization suggests new ecological features of each Se utilization trait. The Sec and Se-cofactor traits favor a host-associated condition, whereas the SeU trait favors aquatic environments. These Se utilization patterns have been verified by comparative metagenomic analysis of distinct bacterial communities from different habitats/environments around the world. The reason for such a trend is not clear yet. One possibility might be that host-associated (such as bacterial parasites in animals) and aquatic (especially marine) environments could provide sufficient supply of Se. However, Se utilization in terrestrial bacteria may be heavily dependent on diverse Se levels in different areas. In addition, the substantial microbial taxonomic diversity in different ecological environments might also explain differences in Se utilization. Surprisingly, we could not identify the strong relationship previously reported between oxygen level and Sec utilization (Zhang *et al.*, 2006; Zhang and Gladyshev, 2010b), as a significant number of both aerobic and anaerobic organisms have this trait. However, considering that the majority of selenoprotein-rich organisms are anaerobic organisms, low oxygen level might, at least in part, contribute to the evolution of new selenoprotein genes. On the other hand, a significant correlation between the Se-cofactor trait and anaerobic conditions demonstrated a strong macroevolutionary trend of this trait for the first time. In the future, it would be important to identify additional ecological factors that influence Se utilization in nature.

In conclusion, we have performed a comprehensive comparative analysis of Se utilization in bacteria, which generated the largest map of Se utilization in this domain. Our data revealed a complex and dynamic evolutionary history for each Se utilization trait. Identification of organisms that only have SelD implies the presence of a new SelD-dependent Se utilization pathway in bacteria. Phylogenetic analyses of key components of Se utilization traits suggested that additional HGT events could be detected for each of them; however, co-transfer of two or more Se traits might be extremely difficult and has not been observed yet. Identification of new selenoprotein-rich phylum and selenoprotein-rich organisms as well as their selenoproteomes may provide important perspectives on the diversity and evolution of different selenoprotein families. Finally, comparative analyses of environmental conditions for all sequenced bacteria and metagenomic samples revealed new and important relationships between ecological environments and Se utilization.

## Figures and Tables

**Table 1:** The list of organisms that contain orphan SelD

*Phyla*	*Organism*
Actinobacteria	*Actinobacterium SCGC AAA015-D07*
	*Actinomadura atramentaria DSM 43919*
	*Actinopolyspora iraqiensis IQ-H1*
	*Amycolatopsis vancoresmycina DSM 44592*
	*Saccharomonospora halophila 8*
	*Saccharomonospora saliphila YIM 90502*
	*Streptomyces ghanaensis ATCC 14672*
Bacteroidetes	*Bacteroidetes bacterium SCGC AAA027-G08*
Cyanobacteria	*Prochlorococcus sp. W7*
	*Prochlorococcus sp. W8*
	*Prochlorococcus sp. W9*
Others	*SAR406 cluster bacterium JGI 0000059-D20*
	*SAR406 cluster bacterium JGI 0000059-E23*
	*SAR406 cluster bacterium SCGC AAA003-E22*
	*candidate division EM 19 bacterium SCGC AAA471-D06*
	*candidate division KSB1 bacterium SCGC AAA252-G07*
	*candidate division KSB1 bacterium SCGC AAA252-N05*
Proteobacteria/alpha/others	*Brevundimonas sp. BAL3*
	*Methylobacterium nodulans ORS 2060*
Proteobacteria/delta	*SAR324 cluster bacterium SCGC AAA001-C10*
	*SAR324 cluster bacterium SCGC AAA240-J09*
Proteobacteria/gamma/Enterobacteriales	*Xenorhabdus bovienii SS-2004*
	*Xenorhabdus nematophila ATCC 19061*
Proteobacteria/gamma/Others	*Gallaecimonas xiamenensis 3-C-1*
Proteobacteria/gamma/Pseudomonadales	*Pseudomonas tolaasii 6264*
Proteobacteria/gamma/Pasteurellales	*Haemophilus aegyptius ATCC 11116*

**Table 2:** New HGT events for each Se utilization trait

*Se trait*	*From (donor)*	*To (recipient)*	*Occurrence of other Se trait in recipient*
Sec	*Microlunatus phosphovorus NM-1* (Actinobacteria/Propionibacterineae)	*Arthrobacter crystallopoietes BAB-32* (Actinobacteria/Micrococcineae)	—
	*Nakamurella multipartita DSM 44233* (Actinobacteria/Frankineae)	*Arthrobacter sp. 162MFSha1.1* (Actinobacteria/Micrococcineae)	—
	*Pseudomonas stutzeri A1501* (Gammaproteobacteria/Pseudomonadales)	*Marinobacter daepoensis DSM 16072* (Gammaproteobacteria/Alteromonadales)	SeU
	*Aeromonas hydrophila pc104A* (Gammaproteobacteria/Aeromonadales)	*Psychromonas sp. CNPT3* (Gammaproteobacteria/Alteromonadales)	SeU
SeU	*Bacillales species* (Firmicutes/Bacillales)	*Lacticigenium naphtae DSM 19658* (Firmicutes/Lactobacillales)	—
Se-cofactor	*Clostridium* species (Firmicutes/Clostridia)	*Fusobacterium sp. 11_3_2* (Fusobacteria)	—
	*Clostridium* species (Firmicutes/Clostridia)	*Treponema phagedenis F0421* (Spirochaetes)	—

Abbreviation: HGT, horizontal gene transfer.

**Table 3:** Distribution of the top 20 selenoprotein families in bacteria

*Selenoprotein family*	*Number of organisms*	*Number of selenoproteins*
Formate dehydrogenase alpha subunit	822	1276
Selenophosphate synthetase	383	394
Glycine reductase complex selenoprotein B	146	261
Glycine reductase complex selenoprotein A	142	165
Proline reductase	110	131
HesB like	86	90
Peroxiredoxin (Prx)	78	91
Coenzyme F420-reducing hydrogenase delta subunit	53	131
Heterodisulfide reductase subunit A	42	103
DsbA-like protein	41	45
Arsenite S-adenosylmethyltransferase	39	41
Selenoprotein W like	35	35
Fe-S oxidoreductase	31	47
Prx-like thiol:disulfide oxidoreductase	28	32
Coenzyme F420-reducing hydrogenase, alpha subunit	26	31
UGSC-containing protein	26	33
ULPU-containing selenoprotein	26	28
Cation-transporting ATPase, E1-E2 family	25	32
Predicted NADH:ubiquinone oxidoreductase, subunit RnfC	25	28
Sulfurtransferase homologous to a rhodanese-like protein	24	25
