# Supplementary material for: Comparative genomics reveals new evolutionary and ecological patterns of selenium utilization in bacteria
Source: ISME J. 2016 Jan 22;10(8):2048–59. doi: 10.1038/ismej.2015.246 (PMC5029168; doi:10.1038/ismej.2015.246)
Supplement: Supplementary Figures S1–S4 [file ismej2015246x1.pdf]

Supplementary figures

Figure S1. Genomic context of *selD* in representative organisms containing orphan SelD. The *selD* gene is highlighted in red. Candidate genes involved in Se/Sec metabolism are shown in different colors. Coding direction is also indicated. A. isochorismatase-like (shown in blue); B. ABC transporter-related ATPase (shown in yellow); C. cysteine desulfurase-like (shown in green).

Figure S2. The fraction of selenoprotein genes in corresponding genomes of completely sequenced organisms. The x-axis represents the total number of annotated genes and the y-axis represents the number of selenoprotein genes in the corresponding genomes.

Figure S3. Relationship between Se utilization traits and environmental factors (oxygen concentration and optimal growth temperature). Organisms are classified into eight groups: Sec trait only, SeU trait only, Se-cofactor trait only, both Sec and SeU traits, both Sec and Se-cofactor traits, both SeU and Se-cofactor traits, Sec and SeU and se-cofactor traits, and no Se utilization traits. (A) Distribution of organisms classified according to their oxygen requirement based on their Se utilization traits. (B) Distribution of organisms classified according to their optimal growth temperature based on their Se utilization traits.

Figure S4. Average fraction of SelD-containing organisms in different environments. The occurrence of *selD* gene was initially identified across a set of metagenomes from freshwater, marine, host-associated, and terrestrial bacterial communities. The average fraction of SelD-containing organisms was then generated after normalizing to a core housekeeping gene *recA*.

Figure S1

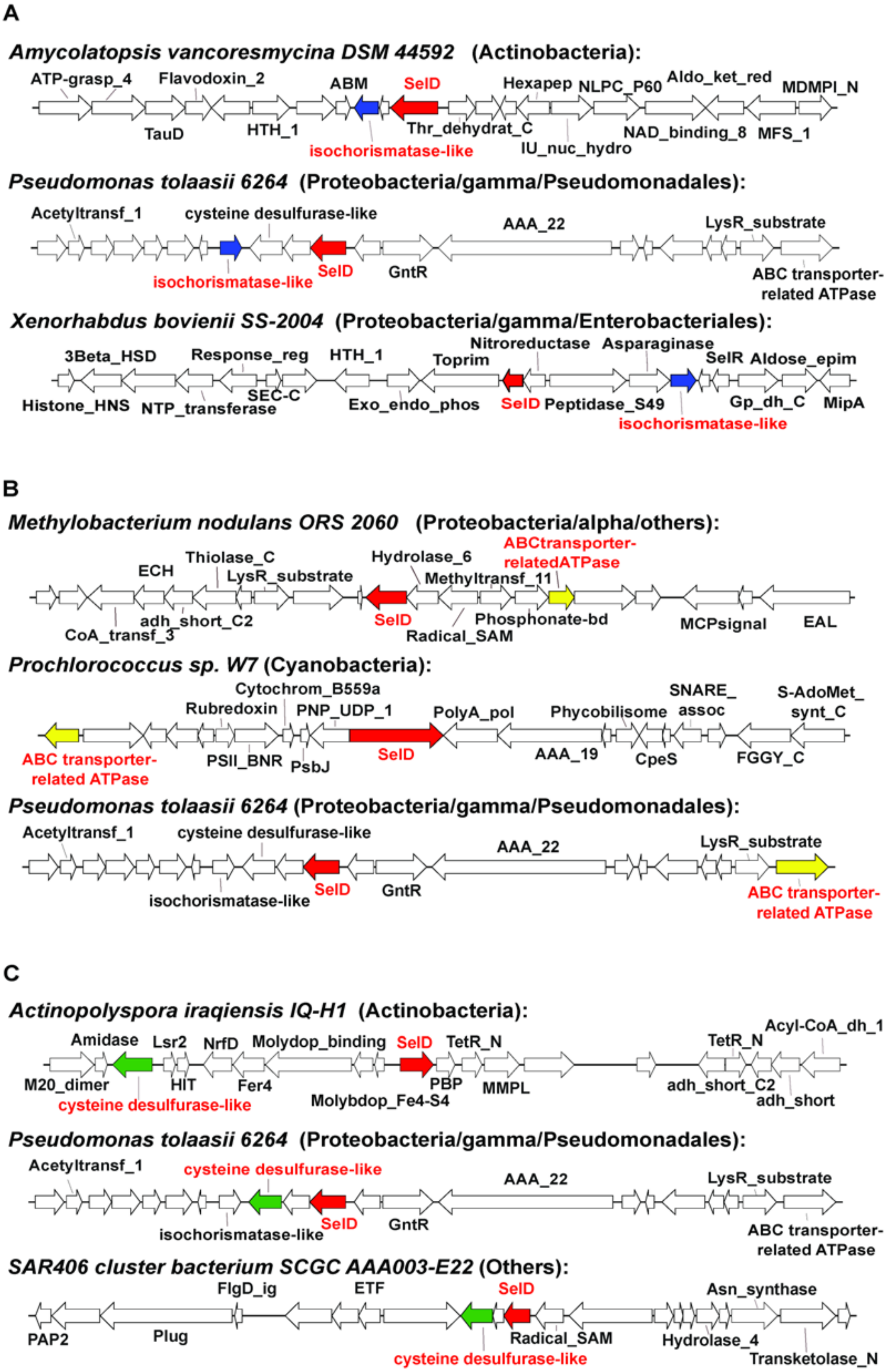
A
Amycolatopsis vancoresmycina DSM 44592 (Actinobacteria):
ATP-grasp_4
Flavodoxin_2
ABM
SelD
Hexapep
NLPC_P60
Aldo_ket_red
MDMPI_N
TauD
HTH_1
Thr_dehydrat_C
NAD_binding_8
MFS_1
isochorismatase-like
IU_nuc_hydro
Pseudomonas tolaasii 6264 (Proteobacteria/gamma/Pseudomonadales):
Acetyltransf_1
cysteine desulfurase-like
AAA_22
LysR_substrate
isochorismatase-like
SelD
GntR
ABC transporter-related ATPase
Xenorhabdus bovienii SS-2004 (Proteobacteria/gamma/Enterobacteriales):
3Beta_HSD
Response_reg
HTH_1
Nitroreductase
Toprim
Asparaginase
SelR
Aldose_epim
Histone_HNS
NTP_transferase
SEC-C
Exo_endo_phos
SelD
Peptidase_S49
Gp_dh_C
MipA
isochorismatase-like
B
Methylobacterium nodulans ORS 2060 (Proteobacteria/alpha/others):
ECH
Thiolase_C
LysR_substrate
Hydrolase_6
ABCtransporter-relatedATPase
Methyltransf_11
adh_short_C2
CoA_transf_3
SelD
Radical_SAM
Phosphonate-bd
MCPsignal
EAL
Prochlorococcus sp. W7 (Cyanobacteria):
Cytochrom_B559a
Rubredoxin
PNP_UDP_1
PolyA_pol
Phycobilisome
SNARE_assoc
S-AdoMet_synt_C
ABC transporter-related ATPase
PSII_BNR
PsbJ
SelD
AAA_19
CpeS
FGGY_C
Pseudomonas tolaasii 6264 (Proteobacteria/gamma/Pseudomonadales):
Acetyltransf_1
cysteine desulfurase-like
AAA_22
LysR_substrate
isochorismatase-like
SelD
GntR
ABC transporter-related ATPase
C
Actinopolyspora iraqiensis IQ-H1 (Actinobacteria):
Amidase
Lsr2
NrfD
Molydop_binding
SelD
TetR_N
TetR_N
Acyl-CoA_dh_1
M20_dimer
HIT
Fer4
PBP
MMPL
adh_short_C2
adh_short
cysteine desulfurase-like
Molybdop_Fe4-S4
Pseudomonas tolaasii 6264 (Proteobacteria/gamma/Pseudomonadales):
Acetyltransf_1
cysteine desulfurase-like
AAA_22
LysR_substrate
isochorismatase-like
SelD
GntR
ABC transporter-related ATPase
SAR406 cluster bacterium SCGC AAA003-E22 (Others):
FlgD_ig
ETF
SelD
Asn_synthase
PAP2
Plug
Radical_SAM
Hydrolase_4
Transketolase_N
cysteine desulfurase-like

Figure S2

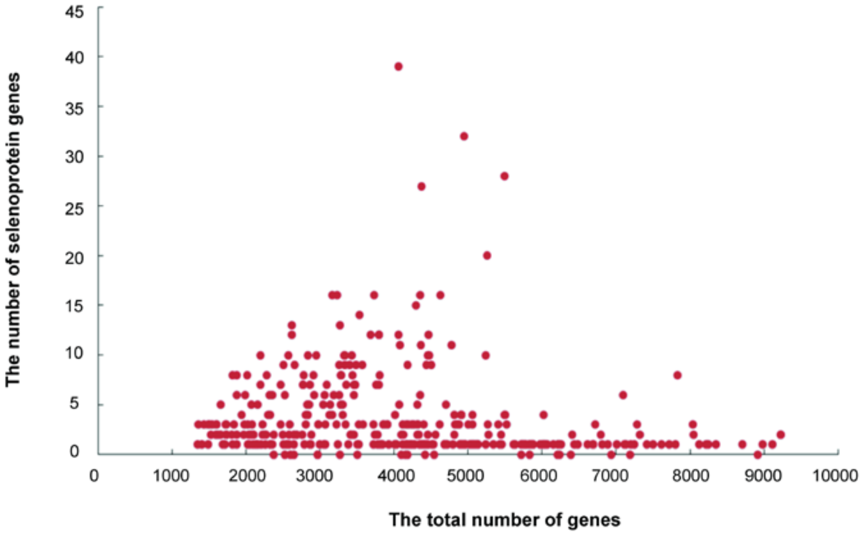

The number of selenoprotein genes
The total number of genes
0
5
10
15
20
25
30
35
40
45
0
1000
2000
3000
4000
5000
6000
7000
8000
9000
10000

Figure S3

A

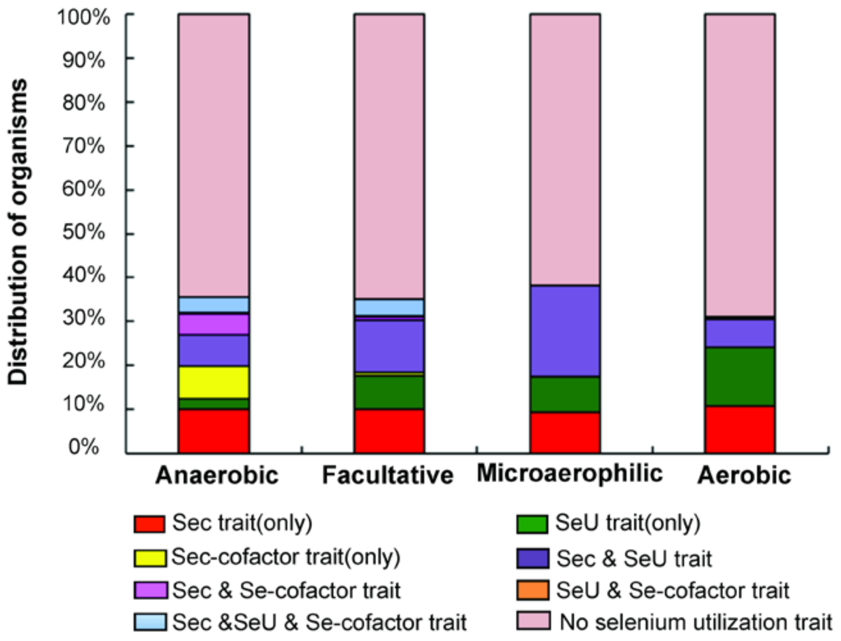

100%
90%
80%
70%
60%
50%
40%
30%
20%
10%
0%
Distribution of organisms
Anaerobic
Facultative
Microaerophilic
Aerobic
Sec trait(only)
SeU trait(only)
Sec-cofactor trait(only)
Sec & SeU trait
Sec & Se-cofactor trait
SeU & Se-cofactor trait
Sec &SeU & Se-cofactor trait
No selenium utilization trait

B

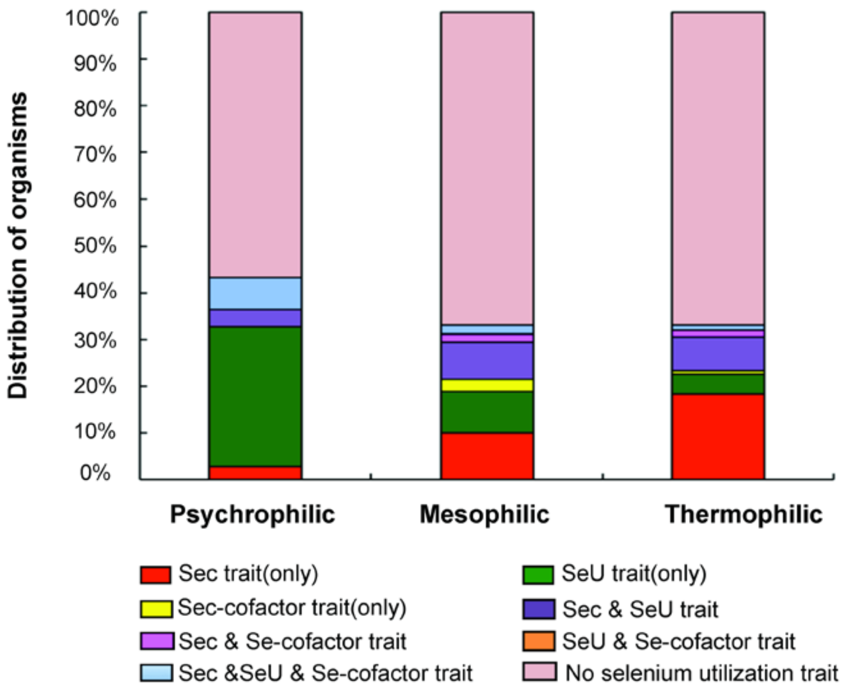

100%
90%
80%
70%
60%
50%
40%
30%
20%
10%
0%
Distribution of organisms
Psychrophilic
Mesophilic
Thermophilic
Sec trait(only)
SeU trait(only)
Sec-cofactor trait(only)
Sec & SeU trait
Sec & Se-cofactor trait
SeU & Se-cofactor trait
Sec &SeU & Se-cofactor trait
No selenium utilization trait

Figure S4

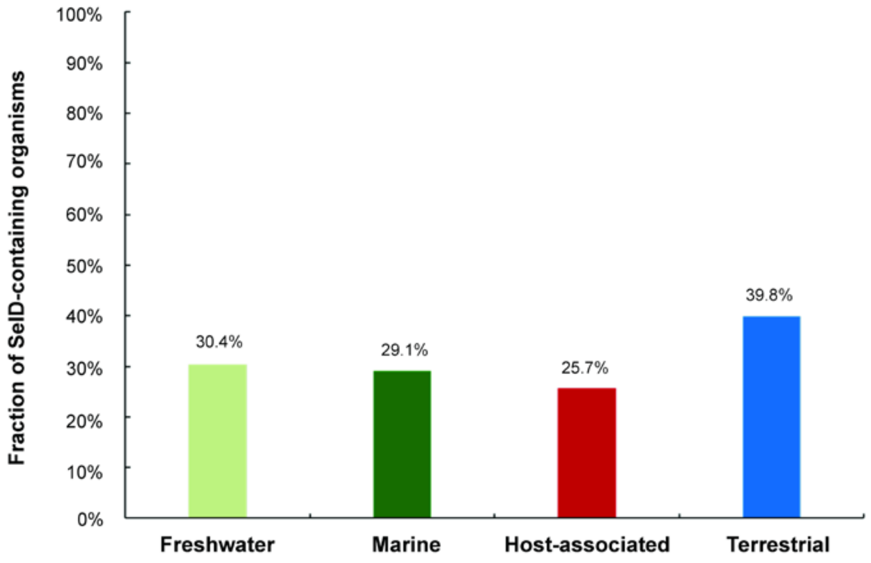
100%
90%
80%
70%
60%
50%
40%
30%
20%
10%
0%
Fraction of SelD-containing organisms
30.4%
29.1%
25.7%
39.8%
Freshwater
Marine
Host-associated
Terrestrial
